# Current state of the art in hypoplastic left heart syndrome

**DOI:** 10.3389/fcvm.2022.878266

**Published:** 2022-10-28

**Authors:** Aditya K. Birla, Sunita Brimmer, Walker D. Short, Oluyinka O. Olutoye, Jason A. Shar, Suriya Lalwani, Philippe Sucosky, Anitha Parthiban, Sundeep G. Keswani, Christopher A. Caldarone, Ravi K. Birla

**Affiliations:** ^1^Laboratory for Regenerative Tissue Repair, Texas Children's Hospital, Houston, TX, United States; ^2^Center for Congenital Cardiac Research, Texas Children's Hospital, Houston, TX, United States; ^3^Division of Congenital Heart Surgery, Texas Children's Hospital, Houston, TX, United States; ^4^Department of Surgery, Baylor College of Medicine, Houston, TX, United States; ^5^Division of Pediatric Surgery, Department of Surgery, Texas Children's Hospital, Houston, TX, United States; ^6^Department of Mechanical Engineering, Kennesaw State University, Marietta, GA, United States; ^7^Division of Pediatric Cardiology, Texas Children's Hospital, Houston, TX, United States

**Keywords:** stem cells, congenital heart defect (CHD), genetics, hemodynamic, regenerative medicine

## Abstract

Hypoplastic left heart syndrome (HLHS) is a complex congenital heart condition in which a neonate is born with an underdeveloped left ventricle and associated structures. Without palliative interventions, HLHS is fatal. Treatment typically includes medical management at the time of birth to maintain patency of the ductus arteriosus, followed by three palliative procedures: most commonly the Norwood procedure, bidirectional cavopulmonary shunt, and Fontan procedures. With recent advances in surgical management of HLHS patients, high survival rates are now obtained at tertiary treatment centers, though adverse neurodevelopmental outcomes remain a clinical challenge. While surgical management remains the standard of care for HLHS patients, innovative treatment strategies continue to be developing. Important for the development of new strategies for HLHS patients is an understanding of the genetic basis of this condition. Another investigational strategy being developed for HLHS patients is the injection of stem cells within the myocardium of the right ventricle. Recent innovations in tissue engineering and regenerative medicine promise to provide important tools to both understand the underlying basis of HLHS as well as provide new therapeutic strategies. In this review article, we provide an overview of HLHS, starting with a historical description and progressing through a discussion of the genetics, surgical management, post-surgical outcomes, stem cell therapy, hemodynamics and tissue engineering approaches.

## Introduction

Hypoplastic left heart syndrome (HLHS) is a congenital heart condition in which a pediatric patient is born with an underdeveloped left ventricle and associated structures (1). This condition affects ~1,000 patients annually in the US (1). If not aggressively treated and managed at the time of birth, HLHS is fatal (1). There have been excellent reviews in recent literature covering specific topics related to HLHS, to include stem cell therapy (2, 3), regenerative medicine approaches (4, 5), tissue engineering strategies (6), and treatment approaches (7). These review articles each provide an excellent overview of a very focused area related to HLHS. The current review serves to provide a comprehensive overview of HLHS, starting with a historical perspective, followed by a review of genetics, stem cell therapy, clinical outcomes, neurodevelopmental aspects, hemodynamics, and proposed tissue engineering therapies.

## Surgical management of HLHS patients

Since the pioneering work by Dr. Norwood and the development of the staged palliative surgical approach (8), high survival rates are now accomplished in tertiary treatment centers (9). HLHS can be diagnosed *in utero* during a routine fetal echocardiography as early as the second trimester and allows for surgical planning at the time of birth (7). At the time of birth, a series of medical management strategies are used to stabilize the patient prior to surgical intervention, as described below.

One of the first tasks is stabilization with prostaglandins to maintain ductal patency. During fetal circulation, the placenta is the primary source of oxygen-exchange with the maternal circulation (10). Since the fetus does not rely on the lungs for oxygen, the ductus arteriosus shunts the blood away from the lungs and provides a flow pathway from the pulmonary artery directly to the descending aorta (10). After birth, this circulation changes and the ductus arteriosus rapidly begins and the pulmonary vascular resistance falls and the neonate now relies on the pulmonary circulation for oxygenation (10). However, in HLHS hearts, prograde aortic flow is minimal or absent and the right side of the heart delivers the majority of the cardiac output to the lungs and through the ductus arteriosus to the body (7). Pharmacologically maintaining patency of the ductus arteriosus with infusion of prostaglandin E1 (PGE-1), a naturally occurring prostaglandin and a known vasodilator, is required to maintain patency of the ductus arteriosus at the time of birth prior to surgical intervention (10).

Once the patient has been stabilized through the use of PGE-1, the next steps include three palliative interventions, the first stage palliation, the second stage palliation and the Fontan procedure (7). Surgical intervention was developed on the philosophy that the right side of the neonatal heart can support both systematic and pulmonary circulation. To achieve this surgically, the right side of the neonatal heart must deliver blood to the lungs for oxygenation and through the systematic circulation to the whole body. A series of staged palliative surgeries were developed to accomplish this task by Dr. Norwood; they were published in his landmark paper in 1983 (8) and were described in an earlier section of this review.

**Figure 1:**
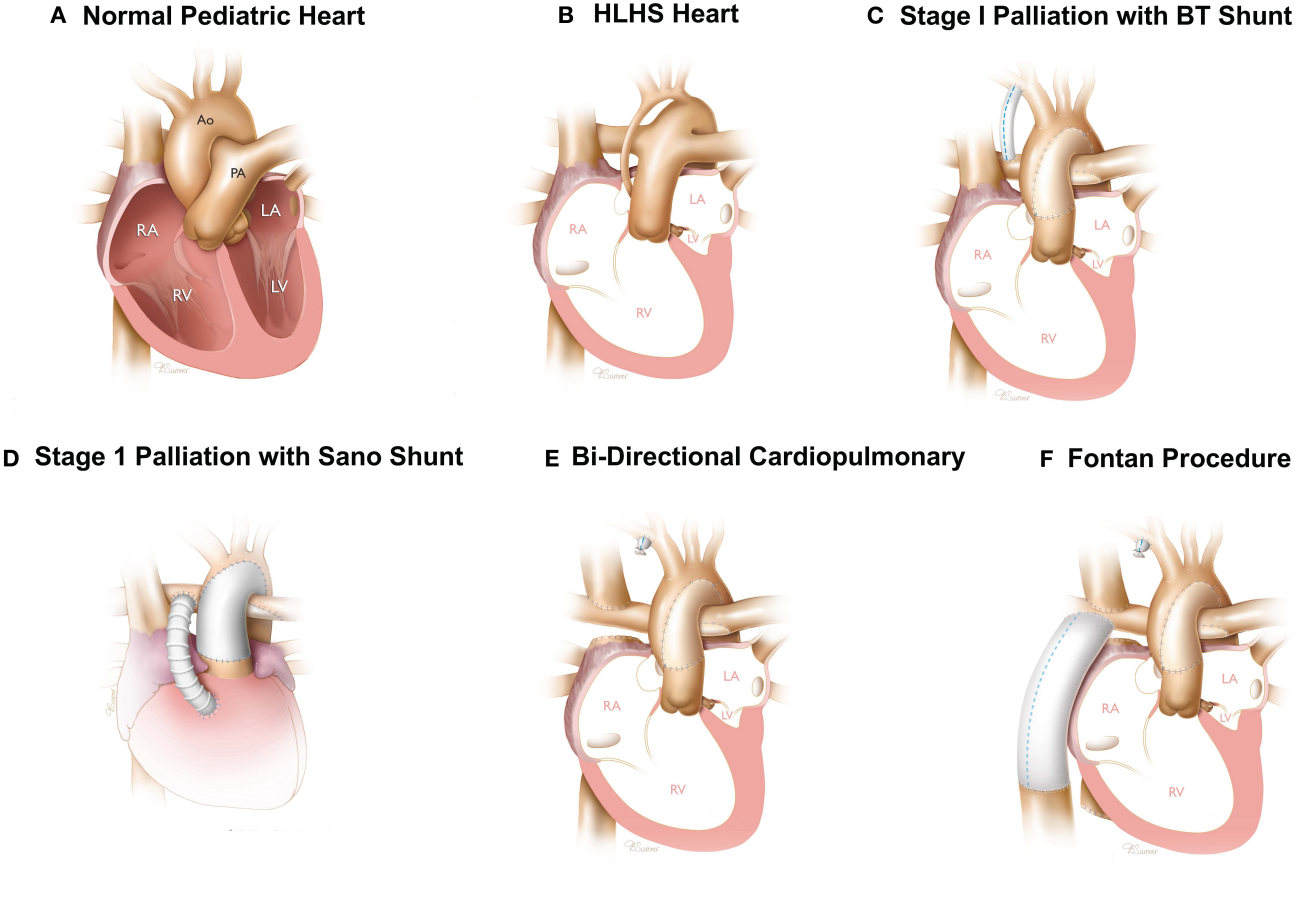
Surgical reconstruction of HLHS hearts—Normal **(A)** and HLHS pediatric heart **(B)**. Three stage surgical reconstruction starting with stage one palliation using a BT shunt **(C)** or sano-shunt **(D)**, bidirectional cavopulmonary shunt **(E)**, and Fontan procedure **(F)**. Images printed with permissions from Texas Children's Hospital.

The characteristics of HLHS is underdeveloped left heart structures including the mitral valve, left ventricle, aortic valve, and ascending aorta and aortic arch (Figures 1A,B), all of which severely limit the ability of the neonatal heart to support systematic circulation (7). The most immediate need is to support both systematic and pulmonary circulation independent of the ductus arteriosus and develop a patent aorta for blood flow through the RV. The four basic objectives of first stage palliation are to: 1. Provide unobstructed systemic cardiac output; 2. Provide a controlled source of pulmonary blood flow; 3. Provide a reliable source of coronary blood flow; 4. Provide unobstructed egress of blood from the pulmonary veins (Figures 1C,D) (8). During the Norwood, the main pulmonary artery is divided amalgamated with the ascending aorta and the reconstructed aortic arch to provide cardiac output to the body and a reliable source of coronary circulation (8). The second objective achieved with the Norwood is to provide a controlled source of pulmonary blood flow which can be provided by a modified Blalock-Taussig-Thomas (mBTTS) shunt, which directs blood from the innominate artery to the pulmonary artery (Figure 1C) (11, 12). An alternative is the Sano shunt, which provides pulmonary blood flow through a conduit between the RV and the pulmonary artery (Figure 1D) (13). These steps result in a parallel circulation where the cardiac output is divided between the systemic and pulmonary circulations. During the Norwood procedure, the atrial septal defect is enlarged to ensure unobstructed egress of oxygenated blood from the left atrium to the right atrium. In addition, the atrial septal defect is enlarged to ensure unobstructed egress of oxygenated blood from the left atrium to the right atrium (14).

The second surgery, known as the bidirectional cavopulmonary shunt, is performed about 4–6 months after the Norwood procedure (15). The objective of the Glenn procedure is to reduce the load on the RV to support both the pulmonary and systematic circulation through the Norwood (15). During the Glenn procedure, the superior vena cava (SVC) is divided from the right atrium and anastomosed directly to the pulmonary artery (Figure 1E) (15). This provides a pathway for venous blood from the upper extremities and head to be directed to the pulmonary circulation directly, thereby unloading the RV of this burden achieved by converting an in-parallel circulation to an in-series circulation. The objective of the second stage procedure is to maintain oxygenation and to convert from an in-parallel to an in-series circulation as an interim step prior to a Fontan procedure.

The third surgery is known as the Fontan (16) and performed 3–4 years after the Glenn procedure. During the Fontan surgery, the inferior vena cava (IVC) is divided and anastomosed to the pulmonary artery using a conduit or a tunnel comprised of a patch and the lateral wall of the right atrium. In both configurations, the physiologic result is inclusion of venous blood flow from the IVC into the pulmonary circulation (Figure 1F) (16).

While the staged palliation surgical management approach is the most common, alternative strategies are also used. A hybrid approach has been used and, in some cases, HLHS patients are directly listed for a heart transplantation, though with the scarcity of donor organs, this strategy becomes challenging. Mechanical support devices have also been used as a bridge to transplantation.

## Genetic basis of HLHS

Genetic studies can be grouped into three categories and are discussed in subsequent sections: (1) Linkage analysis and heritage analysis mapping HLHS to specific regions of the chromosomes. (2) Mutation analysis to identify individual genes responsible for HLHS. (3) Use of induced pluripotent stem cells to elucidate the genetic basis of HLHS.

### Linkage analysis of HLHS patients

Linkage analysis is a powerful tool that identifies the chromosomal location of genes that are responsible for a particular disease (17). This technique has been used in the case of HLHS and several examples are presented here. In one study involving 353 patients, linkage analysis was used to connect the inheritance of bicuspid aortic valve (BAV) dysfunction, a common disorder observed in congenital heart patients, including HLHS, to chromosomes 18q, 5q, and 13q, suggesting the presence of genes whose mutations are responsible for BAV dysfunction (18). Another study, also relying on linkage analysis using 289 patients, demonstrated chromosomal linkage of HLHS abnormalities to chromosome 2p15 (19). Additional studies provided evidence and demonstrated that HLHS was linked to mutations in chromosomes 10q22 and 6q (20) and 21q22.3 (21) and also 11q23 deletion (22). While there is no doubt that linkage analysis has provided valuable insights into the chromosomal locations of mutations related to HLHS, such a strategy has done little to develop a pathway for therapeutic approaches. This strategy was used during an earlier timeframe, prior to the recent and powerful advances in RNA-seq, single-cell seq, and other related strategies that can identify individual genes responsible for HLHS.

### Mutation analysis to identify individual genes responsible for HLHS

Identifying individuals and families of genes responsible for the pathophysiology of HLHS is critical in developing effective therapeutic strategies to treat this patient population. While there is now an abundance of rich literature describing many different gene candidates, the molecular mechanisms leading to HLHS remain poorly studied and understood. One of the earliest reports observed in HLHS patients showed mutations in connexin43 (23). In this study, HLHS patients undergoing heart transplant were analyzed based on PCR analysis of tissue biopsies obtained at the time of transplant. In this group of patients, eight out of 14, or 57.1%, had mutation in connexin43 (23). Given the critical role of connexin43 in intercellular communication, this result was not surprising (24). In addition, NOTCH1 (25–28) and NKX2.5 mutations (29), deletion of ETS1 (30), impaired adrenergic signaling (31, 32), and upregulation of cTnI (33) have all been linked to HLHS. In another study, 87,355 chemically mutagenized mice were screened and whole exome sequencing was performed, identifying 91 recessive mutations in 61 genes that included 34 cilia-related genes and 16 genes involved in cilia transduced cell signaling (34), highlighting the role of the cilia mutations in congenital heart disorders. In yet another recent study, mouse forward genetics was used to link Sap130 and Pcdha9 in mediating left ventricle hypoplasia and increased penetrance of aortic valve abnormalities, both of which are associated with HLHS (35). Changes in micro-RNA expression has been implicated in HLHS (36), including upregulation of miR-486 in certain cases (37). In addition, one study performed a genome-wide exon array analysis to determine differentially expressed genes and alternatively spliced transcripts in the right ventricle (RV) of six neonates with HLHS, compared to the RV and left ventricle (LV) from non-diseased control subjects (38). In HLHS, more than 180 genes were differentially expressed and 1,800 were differentially spliced, leading to changes in a variety of biological processes involving cell metabolism, cytoskeleton, and cell adherence (38).

### HLHS is caused by multiple genes

Recent literature suggests that HLHS is caused by mutations in multiple genes and is not the result of a single mutation only. Examples of genes shown to be cause HLHS include HAND1, GJA1, ZIC3, NKX2.5, NOTCH1, MCTP2, and MYH6 (39). The exact nature of the relative contribution of these genes and how they affect the etiologies observed in HLHS, it is becoming increasing evident that a multitude of genes acting in tanden lead to the anatomical abnormalities seen in HLHS (39).

### Single cell analysis

More recent work has focused on single cell analysis and 3D patch engineering to decipher the role of various genes in HLHS (40). The single cell analysis showed significant changes in many pathways related to cardiomyocyte contraction, heart development, striated muscle differentiation, and cytoskeleton organization. Deficiencies in heart muscle contractility were shown based on work using 3D patches (40). Overall, this work provided a broader perspective of the genetic basis of HLHS showing that a family of genes are responsible, rather than a single gene mutation.

### Induced pluripotent stem cells and genetics of HLHS

Induced pluripotent stem cell derived cardiomyocytes **(**iPSC-CMs) have been used extensively as a disease model in many cardiac disorders (41), though not extensively in the congenital space and much less so in the study of HLHS. In one study, iPSC-CMs were used to demonstrate the role of hypoxia in the development of HLHS through upregulation of the master regulator hypoxia inducible factor (HIF-1α), oncogene-associated cellular senescence, TGF-β1-associated fibrosis and impaired vasculogenesis (42). In a more recent study, whole genome sequencing of iPSC-CMs from three related individuals, identified LDL receptor-related protein LRP2 as a key modulator of cardiomyocyte proliferation and development during embryogenesis, mutagenesis of which could lead to HLHS (43).

### Future studies

The genetic basis of HLHS has been an area of investigation, though a clear signaling pathway that leads to the anatomical malformations of the fetal heart is yet to be established. Understanding the genetic basis of HLHS and the role of hemodynamics is essential to develop effective therapeutic strategies. The current body of literature lacks a cohesive understanding of the genetic basis of HLHS; rather, alterations in the expression of a few select genes are linked to HLHS. The current set of models used to study HLHS development will need to be expanded, and recent advances in bioengineering approaches related to iPSC-CMs and patch engineering will prove to be valuable in these studies.

iPSC-CMs technology has not been leveraged to its full potential as a tool to significantly understand the key modulators of HLHS. The main advantage of iPSC-CMs is the ability to obtain patient-specific information on the genetic modulators of HLHS. In addition, iPSC-CMs can be subjected to known master regulators of HLHS, like volume overload and cyanosis, and the responsiveness of these cells can be characterized in an isolated *in vitro* monolayer culture system. The ability to bioengineer three-dimensional heart muscle from these cells further adds to the advantages of this technology as the genetic modulators and response to external stimuli can now be investigated in three-dimensional tissue (44).

## HLHS outcomes

Table 1 includes a list of reports describing 1-year mortality after the Norwood procedure (9, 45–57). Scanning Table 1 provides some insight into post-surgical outcomes for HLHS patients. A review of the data shows large variations in outcomes with no clear trends. The range of mortalities is broad, from a low of 15% from 157 patients during the period 1996–2007 at University Hospital Schleswig-Holstein in Germany (52) to a high of 60% from 129 patients during the period 1983–2004 at Royal Children's Hospital in Australia (50). Based on an analysis of the outcomes data presented in Table 1, the HLHS patient survival 1 year after stage one palliation surgery has improved significantly in the current decade compared with survival 1990–2010. Further advances in management of HLHS include stem cell therapies and bioengineering solutions, which have the potential to increase survival of HLHS patients. While surgical management will continue to be the standard of care for HLHS patients in the near-term, use of innovative therapies (stem cell therapy and bioengineering) are essential tools in increasing patient survival and improving outcomes.

**Table 1:** Outcomes table.

**ID**	**Year** **published**	**Senior author**	**Country**	**Hospital**	**# of patients**	**Years**	**1-yr. mortality**	**References**
1	1997	Quaegebeur	USA	Columbia University	53	1990–1996	40.0%	(45)
2	2001	Anderson	UK	Guy's Hospital	64	1995–2000	48.0%	(46)
3	2002	Klitzner	USA	Multicenter	1,986	1998–1997	40.9%	(47)
4	2005	Vogt	Germany	University Hospital Muenster	41	1992–2002	29.0%	(48)
5	2006	Ohye	USA	University of Michigan	111	2001–2003	21.0%	(9)
6	2006	Brawn	UK	Birmingham Children's Hospital	333	1992–2004	29.0%	(49)
7	2007	Wlikinson	Australia	Royal Children's Hospital	129	1983–2004	60.0%	(50)
8	2008	Ishino	Taiwan	National Taiwan University Hospital	62	1998–2007	20.0%	(51)
9	2009	Kramer	Germany	University Hospital of Schleswig-Holstein	157	1996–2007	15.0%	(52)
10	2011	Jennifer Li	USA	Multicenter	2,557	2000–2009	22.0%	(54)
11	2012	Latal	Switzerland	University Children's Hospital	31	2004–2008	36.0%	(53)
12	2014	Krasemann	UK	Evalina London Children's Hospital	138	2005–2011	41.4%	(55)
13	2018	Skalski	Poland	Jagiellonian University Children's Hospital	85	2007–2011	28.2%	(56)
14	2019	Spray	USA	Children's Hospital of Philadelphia (CHOP)	1,663	1984–2014	25.9%	(57)

### HLHS cavopulmonary hemodynamics

The selection of the optimal shunt during the Norwood procedure and the construction of the optimal cavopulmonary connection configuration during the Glenn and Fontan surgeries rely on multiple patient-specific factors such as RV function, cardiac and vessel anatomies, inflow conditions and respiration rates, and their relations with the systemic and pulmonary flow dynamics generated at each surgical stage (58). Experimental approaches based on state-of-the-art *in vitro* flow diagnostic techniques, as well as computational fluid dynamics (CFD) strategies combining patient-specific flow models with lumped parameter networks have been successfully implemented to improve surgical planning in HLHS patients (59). As a result, the bulk of the literature published to date on HLHS hemodynamics has mostly focused on the extracardiac hemodynamics generated by the different palliative surgical stages and the use of this knowledge in surgical planning. In contrast, the pre- and post-surgical intracardiac hemodynamics of the single RV, which are key predictors of HLHS patient survival (60), remain largely unexplored. This section describes the important contributions made to the characterization of HLHS hemodynamics to date.

#### Norwood hemodynamics

While post-operative complications of the Norwood procedure may be linked to pre-operative patient characteristics (e.g., presence of non-cardiac/genetic abnormalities, weight <2.5 kg, right dominant single ventricle) (61), the specific hemodynamics and flow resistance of the systemic-pulmonary arterial shunt has also been suggested as an important prognostic factor for patient survival. As demonstrated in an early *in vitro* study, which evaluated pressure-flow relationships in systemic-to-pulmonary Blalock-Taussig shunts of different diameters, an increase in shunt diameter resulted in reduced pressure gradients at both the proximal and distal anastomoses (62). More recently, a computational parametric investigation conducted in an idealized Blalock-Taussig shunt geometry revealed that not only shunt diameter but also anastomosis placement was critical to oxygen delivery to both systemic and coronary circulations (63). Lastly, multiscale computational flow modeling has also been used to compare the hemodynamic performance of different shunt configurations. The simulations suggested that the Sano shunt consistently generated more favorable hemodynamics (i.e., lower RV systolic and diastolic pressures, lower pulmonary-to-systemic flow ratios, and higher coronary perfusion pressure).

The surgical management of HLHS consists of redirecting the venous deoxygenated blood toward the lungs while bypassing the RV *via* a staged surgical approach. A challenge raised by the construction of this bypass is the requirement to achieve a balanced flow split between both lungs while minimizing flow energy loss. The dominant objective in single ventricle management is to achieve the Fontan circulation, which depends upon kinetic energy to propel blood through the lungs without a sub-pulmonary ventricle. The concept of energy is critical to the success of this staged palliative surgery. In fact, the single RV must eject blood with sufficient kinetic energy to overcome energy losses caused by the viscous friction along the entire vasculature and the hemodynamic disturbances (e.g., mixing, collision, separation, and recirculation) generated at each stage of the surgical cardiac and vascular reconstructions (64). Therefore, optimization of the staged surgery is critical to produce efficient hemodynamics necessary for positive long-term clinical outcomes (65).

#### Glenn and Fontan hemodynamics

The hemodynamics of the Glenn and Fontan surgical reconstructions have been documented in many *in vitro* and CFD studies. *In vitro* measurements in realistic glass models mimicking a bidirectional Glenn cavopulmonary connection suggested the benefits of this connection over a dilated atriopulmonary connection by demonstrating its ability to reduce fluid energy dissipation, achieve physiologic distribution of total flow, and maintain some hepatic venous flow to each lung (66). Pulsatile flow simulations in hemi-Fontan and bidirectional Glenn geometries reconstructed from magnetic resonance, angiocardiogram, and echocardiogram anatomic data complemented the previous experimental findings by indicating no substantial difference in power loss and flow distribution to each lung (67).

However, the bulk of the literature published to date on Glenn and Fontan hemodynamics has focused on the identification of key geometrical parameters affecting the hydraulics of the Fontan total cavopulmonary connection (TCPC). Experimental particle image velocimetry measurements in realistic stereolithography intra-atrial connection models have revealed the existence of complex, unsteady, and highly three-dimensional flow structures, suggesting a substantial degree of energy loss in this type of connection (68). Those results are supported by 4D-flow MRI and patient-specific CFD studies, which have evidenced the existence of high vorticity magnitudes and atrial recirculation in the intra-atrial connection (69), and the hydraulic superiority of the lateral tunnel Fontan operation relative to any other method (67).

Another important finding suggested by the literature on TCPC hemodynamics is the stronger dependence of energy loss on the TCPC topology and geometrical features than on the Fontan surgical option. In fact, a retrospective analysis of CFD and *in vitro* flow data revealed that the minimum cross-sectional area of the pulmonary arteries at the TCPC outlets was a stronger predictor of energy loss characteristics than the surgical procedure (extra- vs. intra-cardiac conduit) (70). Another CFD analysis using realistic pulsatile flow boundary conditions and TCPC geometries featuring extra- and intra-cardiac conduits demonstrated that power dissipation was primarily influenced by the actual cross-sectional area of the inferior vena cava anastomosis (71). Lastly, steady flow pressure measurements and flow visualization conducted in idealized TCPC glass models suggested that caval offsets and anastomotic flaring could reduce the hydraulic power loss by half relative to no offset and by at least 45% relative to no flaring, respectively (72, 73). Altogether, those studies suggest that patient-specific flow modeling could be an effective surgical planning tool toward the improvement of HLHS patient outcome and the reduction of surgical risks (74).

#### RV hemodynamics during staged palliation

While the re-engineering of the right side of the heart is a critical requirement to support both pulmonary and systematic circulations in HLHS patients (7), RV function is a key determinant of HLHS patient survival (60). Mathematical and computational approaches with various degrees of sophistication have been proposed to estimate RV hemodynamics and function in HLHS. Flow simulations in a post-Norwood RV geometry confirmed the hemodynamic superiority of the Sano shunt over the Blalock-Taussig shunt, as suggested by the predicted reduction in RV workload and no substantial difference in systemic blood flow (75). Another computational model based on patient-specific RV anatomies reconstructed from magnetic resonance imaging revealed the strong dependence of interventricular pressure gradients, filling dynamics and capacity on RV shape and temporal deformation patterns (76). To date, the only quantification of the native pre-surgical HLHS RV hemodynamics was performed numerically in a fetal heart in the context of cardiac development (77). This investigation, which assessed fetal blood flow using 4D spatiotemporal image correlation ultrasound and numerical modeling, revealed that despite a larger right-ventricular cavity size and a greater cardiac output, HLHS fetal hearts generated essentially the same global interventricular hemodynamics as a normal heart.

#### Future studies

The synergies suggested by previous RV studies between interventricular RV flow dynamics, cardiac function, and TCPC power loss suggest that the knowledge of pre- and post-surgical RV hemodynamics on a patient-specific basis could guide clinical decision making and promote patient survival (78). However, determining the exact role played by RV hemodynamics in the long-term outcome of the right heart surgical reconstruction requires the assessment of the hemodynamic alterations experienced by the RV during the course of the staged Fontan palliation. While this knowledge gap has not been addressed yet, computational strategies have been designed and successfully implemented to assess the potential mechanical changes of other cardiac defects such as discrete subaortic stenosis (DSS) (79–81). Cine-magnetic resonance imaging data was used in tandem with state-of-the-art fluid-structure interaction modeling to predict native blood flow patterns in patient-specific left-ventricular models featuring normal and DSS-prone outflow tract anatomies, and to characterize the resulting myocardial mechanical stresses. A similar modeling strategy could be deployed to elucidate RV hemodynamics in patient-specific post-Norwood RV anatomies, and to identify particular blood flow patterns and mechanical stresses associated with good clinical outcomes.

### Stem cell therapy in HLHS patients

Stem cell therapy is conceptually based on the idea of delivery of isolated cells to the region of injury in an attempt to promote repair and/or regeneration (82). Stem cell therapy has been evaluated in many different fields, particularly in the realm of myocardial infarction in the adult population (83). In comparison, there is a much smaller, though growing body of literature, with varying degree of success, in the pediatric congenital heart field, particularly related to HLHS patients (84–89). Important considerations in stem cell therapy are the source of cells, bone marrow derived mesenchymal stem cells (BMMSCs) being a common choice, mode of delivery (intra-muscular vs. intra-coronary), the number of cells, and timing of stem cell delivery relative to disease progression. Our discussion on this topic is divided into the following sections: (1) lessons learnt from stem cell therapy in adult heart patients, (2) stem cell therapy for pediatric HLHS patients, (3) summary and future perspective.

#### Lessons learnt from stem cell therapy in the adult heart

Stem cell therapy for myocardial infarction in adult hearts is now a large and expansive field, with numerous lessons being learnt (82). However, our goal is to focus this discussion on the potential mode of action of injected cells. How are the injected cells acting on the host tissue to provide a functional benefit? Initially, it was hypothesized that injected cells would integrate with host myocardium, transform to become contracting cardiomyocytes, electromechanically couple with host cardiomyocytes, and provide direct functional improvement to an otherwise failing heart (82). Conceptually, this was the basis of the field and if realized, would truly be revolutionary in the field of adult cardiac regeneration. However, this was not the case for many reasons, perhaps the most significant of which was the low rate of local cell retention: <5% of injected cells were retained at the site of injury (90). Irrespective of the challenges in the field, many lessons have been learnt regarding the potential mode of action of injected stem cells. It is now hypothesized that injected stem cells act through one of several mechanisms, including the release of paracrine signaling factors, promoting neovascularization and/or recruitment of resident or circulating stem cells, all of which serve to either increase the number of functional cardiomyocytes or promote neovascularization (90). With such a rich literature in the adult cardiac stem cell transplantation space, it provides a strong background to initiate similar studies in the pediatric congenital cardiac space, particularly related to HLHS.

#### Stem cell therapy for pediatric HLHS patients

Many of the factors discussed earlier apply to stem cell therapy for HLHS patients, to include the source and number of cells and mode of delivery. However, specific to HLHS patients is the timing of stem cell delivery. The surgical palliation of HLHS is a complex three-stage process, progressing through the Norwood, Glenn, and Fontan surgeries. Therefore, the timing of stem cell delivery becomes crucial and most studies making use of stem cell therapy have been at the time of stage two palliation.

Table 2 provides an overview of several studies describing stem cell therapy in HLHS patients, and these are discussed in subsequent sections. Table 2 is designed to serve as a survey of the recent literature on stem cell therapy in HLHS, rather than an exhaustive list of all published studies. Earlier studies in 2010 (89) and 2015 (87) were designed to provide safety of injected cells in single HLHS patients at the time of the Glenn surgery, followed by the Phase I TICAP trial in 2015 (87) and Phase II PERSEUS trial in 2017 (86), all of which are discussed below.

**Table 2:** Stem cell therapy.

**Year**	**Senior author**	**Cell type**	**Number of cells**	**Mode of delivery**	**Number of patients**	**Time of delivery**	**RV EF**	**References**
2010	Schranz	BMMSCs	Not specified	Intracoronary	1	Glenn	44% after 14 months	(89)
2015	Nelson	UCB mononuclear cells	18 million cells	Intramyocardial	1	Glenn	35–50%	(84)
2015	Oh	CDCs	3.5 × 10^5^ cells per kg of body weight	Intracoronary	14, 7 with cells, 7 controls	Glenn and Fontan	46.9%+/-4.6% to 52.1%+/-2.4%	(87)
2017	Oh	CDCs	3.5 × 10^5^ cells per kg of body weight	Intracoronary	41	Glenn and Fontan	35.3%+/-9.2% to 41.7%+/-7.4%	(86)
2017	O'Leary	BMMNCs	2 × 10^6^ cells per kg of body weight	Intracoronary	1	As an Adult	35–40% after 6 months	(88)
2019	Nelson	UCB mononuclear cells	0.1 ml per kg of body weight	Intramyocardial	10	Glenn	No change	(85)

One of the earlier case studies was described in 2010, in which intracoronary injection of bone marrow mesenchymal stem cells (BMMSCs) during stage II procedure in a single pediatric HLHS patient proved to be safe (89). This was an initial proof of concept study to demonstrate the feasibility of BMMSCs at the time of Glenn, without much detail to any potential functional benefit and/or mode of action. While the results of this study demonstrated safety of BMMSCs delivery in a single HLHS patient at the time of Glenn, many unanswered questions remained. A second case study was published in 2015, using mononuclear cells from umbilical cord blood, again injected during stage II surgery, increasing RV ejection fraction from 30–35 to 50% (84).

Phase I results of the TICAP trial were published in 2015, making use of intracoronary infusion of cardiac progenitor cells (CPCs) in 14 patients, seven undergoing staged palliation with CPCs infusion and an equal amount with only staged palliation (87). Patients treated with CPCs showed RV ejection fraction improvement from baseline to 3-month follow-up (46.9 ± 4.6% to 52.1 ± 2.4%; *P* = 0.008) (87). Compared with controls at 18 months, cardiac MRI analysis of CPC-treated patients showed a higher right ventricular ejection fraction (31.5 ± 6.8% vs. 40.4 ± 7.6%; *P* = 0.049) (87). This was followed up by a Phase II clinical trial, PERSEUS with 41 patients; at 3 months, the absolute changes in ventricular function were significantly greater in the CPC-treated group than in the controls (35.3%+/-9.2% to 41.7%+/-7.4%; *P* = 0.0002) (86).

Another recent study made use of autologous bone marrow derived mononuclear cells administered *via* cardiac catheterization to the coronary circulation in a single patient 23 years after the Fontan Surgery, showing decrease in ventricular size 3 months after cell injection (88).

A more recent study published in 2019 showed the feasibility of intramyocardial injections of mononuclear cells derived from umbilical cord bloods in a Phase I clinical trial with 10 patients, though there were no functional benefits resulting from this intervention (85).

#### Summary and future perspective

Stem cell therapy in HLHS patients is at a stage of infancy, and very few studies have been conducted in a very small patient population. The mode of delivery has been either intracoronary or intramyocardial, and the number and source of cells has varied. The time of delivery has been consistent, with most studies conducted at the time of the second stage palliation. Source and number of cells, along with the timing of delivery, must be optimized, and the modes of action must be elucidated. Furthermore, a combinational therapy consisting of stem cells coupled with growth factors to increase the contractile function of the RV may also prove to be beneficial.

### Tissue engineering and regenerative medicine

Recent advances in bioengineered functional cardiovascular tissue have provided a novel opportunity for new technologies, both to increase our understanding of HLHS and to provide therapeutic options. The definition of tissue engineering has been very elegantly presented in a recent publication (91): Tissue engineering is a multidisciplinary field bringing together experts from engineering, life sciences and medicine, utilizing the building blocks of cells, biomaterials and bioreactors for the development of three-dimensional artificial tissue and organs which can be used to augment, repair and/or replace damaged and/or diseased tissue. Or in simpler terms, tissue engineering is the field focused on fabricating three-dimensional tissue, and applied to the cardiac space, this includes heart muscle (92), ventricles (93), Purkinje networks (94), and whole hearts (95).

The tissue fabrication process has been presented in a very methodological manner in a recent publication (96). In summary, iPSCs are generated from peripheral blood mononuclear cells, isolated from a routine blood draw and converted to cardiomyocytes (iPSC-CMs), using an established protocol (97). iPSC-CMs are cultured within a 3D matrix, resulting in the formation of contractile heart muscle tissue (92). Recent developments in the field of 3D bioprinting have provided a powerful tool to fabricate patient-specific tissue, to perfectly fit the geometry of the defect or to match the patient's anatomy (91). Electromechanical stimulation is used for the maturation and development of iPSC-CMs (98), and subsequent vascularization of the bioengineered tissue is required to support metabolic activity.

While presented in a very simple manner, fabricating cardiovascular tissue is not without its challenges. Some of the challenges include the inability to generate large numbers of mature iPSC-CMs, fabricating heart muscle with high contractility, and optimized bioreactors for electromechanical stimulation. Recent advances point to the development of highly functional 3D cardiac patches, though functional integration with host tissue remains a challenge.

There are two potential applications of bioengineered cardiovascular models in HLHS: (1) tools to understand the underlying molecular mechanisms and (2) potential therapeutic strategies, as detailed in a recent review (6).

#### Tools to understand the underlying molecular mechanisms in HLHS

As one example, there is a high incidence of progressive myocardial dysfunction after the Norwood surgery, as discussed in an earlier section. The goal is to identify molecular indicators responsible for myocardial dysfunction. To accomplish this, blood samples from patients at the time of Norwood can be used to generate iPSCs and cellular difference can be used to correlate with observed clinical outcomes. These iPSCs can further be used to bioengineer 3D patches and that can be used to model tissue level contractility in post-surgical response of HLHS patients. Bioengineered right ventricles (93) can be used to study the effect of volume overload on heart muscle function and subsequent myocardial dysfunction. iPSCs-CMs, patches and ventricles can be used as powerful tools to provide mechanistic information at the cell, tissue, and organ levels.

#### Potential therapeutic strategies

Bioengineered models may someday be used as potential therapies to support HLHS hearts. As an example, biological contractile pumps (99) can be used in the Fontan circuit to provide pulsatile support for blood, as opposed to the inert grafts currently in use which only serve a passive role. These biological pumps can support the flow of blood through the Fontan circuit. Another example is the use of bioengineered heart muscle, which can be used to add musculature to the right ventricle, thereby augmenting contractile function to support the increased blood volume after the Norwood surgery. Heart transplantation is another important therapeutic approach, but the current scarcity of donor hearts precludes heart transplant as routine management strategy. However, in the future, if bioengineered hearts become available, heart transplantation may be expanded as a treatment option for all HLHS patients, or at minimum, expanded to patients considered high risk for palliative surgeries.

## Summary

Our understanding of HLHS has increased over the past two decades, including recent advances in stem cell therapy, dissecting the genetic basis of HLHS, and management of progressive myocardial dysfunction using ventricular assist devices and heart transplantation. Recent advances in tissue engineering and regeneration provide promise to develop new therapies based on stem cell therapy and bioengineered cardiovascular tissue, including patches, ventricles, biological pumps, and whole hearts.

The goal of new therapies is to improve the survival of HLHS patients. This can be accomplished based on an understanding of the genetic basis of the disease, as the master regulator(s), once identified, can be blocked or chemically inhibited. Furthermore, based on an understanding of the molecular basis predictive tools can be developed to quantify the risk of mortality for HLHS patients. Tissue engineering tools, are very powerful, and can be used to increase our understanding of the basic mechanisms and can also be used for repair of the HLHS heart and in the future, bioengineered hearts can be used for HLHS patients and alleviate current shortage of donor hearts.

## Author contributions

AB prepared the section on historical perspective, introduction, and abstract. WS, OO, and CC prepared the section on surgical management of HLHS. SL prepared the introduction. RB the prepared sections on genetic basis of HLHS, tissue engineering, regenerative medicine, and summary. SL and SK prepared section on HLHS outcomes. PS and JS prepared the section on hemodynamics. AP the prepared section on stem cell therapy in HLHS patients. All authors contributed to the article and approved the submitted version.

## Conflict of interest

The authors declare that the research was conducted in the absence of any commercial or financial relationships that could be construed as a potential conflict of interest.

## Publisher's note

All claims expressed in this article are solely those of the authors and do not necessarily represent those of their affiliated organizations, or those of the publisher, the editors and the reviewers. Any product that may be evaluated in this article, or claim that may be made by its manufacturer, is not guaranteed or endorsed by the publisher.
